# Short-chain fatty acid administration via water acidifier improves feed efficiency and modulates fecal microbiota in weaned piglets

**DOI:** 10.1093/jas/skab307

**Published:** 2021-10-22

**Authors:** Mandy M Lingbeek, Klaudyna Borewicz, Erica Febery, Yanming Han, John Doelman, Sandra J A van Kuijk

**Affiliations:** 1 Trouw Nutrition R&D, P.O. Box 299, 3800 AG, Amersfoort, The Netherlands; 2 Drayton Animal Health Ltd, Alcester Road, Stratford-on-Avon, Warwickshire CV37 9RQ, UK

**Keywords:** growth performance, microbiota, short-chain fatty acids, water acidifier, weaned piglets

## Abstract

This study examined the effect of a water acidifier containing free and buffered short-chain fatty acids (**SCFA-WA**) on growth performance and microbiota of weaned piglets. In total, 192 male piglets, approximately 4 wk of age, were allocated to 24 pens (12 per treatment) with 8 piglets per pen. The piglets received either regular drinking water (negative control) or drinking water with the acidifier supplied at 2 L/1,000 L. Body weight and feed intake were measured weekly on pen level. During the first 2 wk, daily visual assessment and scoring of the feces was conducted. Fecal samples of three piglets per pen were collected on days 14 and 42 for high-throughput sequencing analysis of the microbiota. Piglets offered SCFA-WA had significantly improved feed efficiency in the third week (*P* = 0.025) and over the whole study period (days 0 to 42, *P* = 0.042) compared with piglets in the negative control group, with a strong tendency observed during the first feeding phase (days 0 to 21, *P* = 0.055). Furthermore, the water acidifier group had a higher water intake than piglets provided with control water during the second feeding phase (days 21 to 42, *P* = 0.028) and over the whole study period (days 0 to 42, *P* = 0.043). There was no significant difference in body weight, average daily gain, or average daily feed intake (days 0 to 21, 21 to 42, 0 to 42). Furthermore, there was no overall significant difference in fecal scoring between the treatments. In terms of the fecal microbiota response, piglets offered the water acidifier showed a significantly higher relative abundance (**RA**) of genus *Clostridium sensu stricto 1* and a lower RA of genus *Streptococcus* compared to the control. Furthermore, the redundancy analysis showed a positive association between improved feed efficiency and daily weight gain and RA of *Butyricicoccus* and *Faecalibacterium*. In conclusion, consumption of the water acidifier containing free and buffered SCFA modulated the microbiota and improved feed efficiency in piglets.

## Introduction

The weaning process is a stressful time for piglets, as they adapt to new diets and transition to new physical and social environments. During the weaning phase, piglets often have reduced feed intake, subsequently leading to inadequate utilization of nutrients in terms of both digestion and absorption, and an increased incidence of diarrhea (Suiryanrayna and Ramana, 2015; Gresse et al., 2017; Nowak et al., 2021). Recent data indicate that in response to the stress of weaning, only 50% of piglets eat in the first 24 h postweaning, whereas approximately 10% take longer than 48 h before the consumption of a meal (Heo et al., 2013; Nowak et al., 2021). The transition of weaning also coincides with increased stomach pH (Heo et al., 2013; Suiryanrayna and Ramana, 2015; Nowak et al., 2021). This factor may compromise the activity of pepsin, an enzyme secreted into the stomach to aid in protein digestion and which functions optimally in a pH range of 2.0 to 3.5 (Partanen and Mroz, 1999; Heo et al., 2013; Nowak et al., 2021). When digestion is reduced, fewer nutrients are available for absorption by the animal and over the course of several days could lead to a reduction in growth (Suiryanrayna and Ramana, 2015; Nowak et al., 2021). Lower pepsin activity leads to a higher availability of undigested proteins available for fermentation in the gut by the resident microbiome. Increased intestinal protein fermentation and pH are often linked to increased incidence of diarrhea (Pieper et al., 2012; Gilbert et al., 2018; Nowak et al., 2021). Under stable conditions, low stomach pH also functions as a barrier against (possibly pathogenic) microbes (Partanen and Mroz, 1999; Heo et al., 2013; Nowak et al., 2021). Thus, an increased stomach pH could allow more microbes to enter and colonize the intestines (Ravindran and Kornegay, 1993; Tugnoli et al., 2020). Lastly, the low pH of the stomach also stimulates the pancreatic secretions of digestive enzymes and bicarbonate (Partanen and Mroz, 1999; Nowak et al., 2021).

Reduction of stomach pH through the use of acidifiers added via feed or drinking water can have beneficial effects on both growth performance and microbiota of weaning piglets (Partanen and Mroz, 1999; Nowak et al., 2021). Acidifiers also have an inhibitory effect on microbes present in the feed and water, in addition to providing buffering capacity and improvements in smell and palatability of feed (Nowak et al., 2021). Next to the acidifying effect in the stomach, the organic acid component also serves as an energy source for the gastrointestinal tract epithelia and are known to have a strong inhibitory effect on bacteria (Suiryanrayna and Ramana, 2015; Nowak et al., 2021), particularly the gram-positive strains due to their structure (Suiryanrayna and Ramana, 2015). Acidifiers often contain short-chain fatty acids (**SCFA**), which are known to have beneficial effects for the host (Mroz, 2005). The SCFA, including formic acid, acetic acid, propionic acid, and butyric acid, are produced via microbial fermentation in the intestines and play a major role in gut health (Xiong et al., 2019). These SCFA can alter the pH of the gastrointestinal tract, increase epithelial proliferation, affect gastric emptying, digestibility of nutrients, pancreatic enzyme secretion, and the microbiota (Mroz, 2005; Xiong et al., 2019). Additionally, SCFA have been shown to have an effect on pig health and performance (Mroz, 2005). However, our understanding of the effect of water acidifiers on growth performance, health, and in particular gut microbiota in weaned piglets is still limited. Accordingly, the objective of the present study was to determine the effects of a water acidifier containing free and buffered SCFA on growth performance and microbiota of weaned piglets.

## Materials and Methods

### Ethics approval

The study was approved by the DAH Animal Welfare and Ethical Review Board (AWERB) prior to animals being sourced and was carried out under Home Office licence. The study was discussed and approved by the AWERB prior to the start of the in-life phase.

The farm housing and husbandry were representative of EU farming conditions and met relevant ethical, hygienic, and animal welfare requirements.

### Animals and dietary treatments

In total, 192 male piglets (Large White × Landrace × Duroc) at an average live weight of 8.73 kg (between 6.6 and 11.6 kg) and approximately 4 wk of age were housed at Drayton Animal Health Ltd. (DAH, Stratford-upon-Avon, UK). The newly weaned piglets were randomly allocated to 24 pens, in groups of 8 piglets, blocked by weight, equally divided over 3 rooms. The pens were randomly allocated to two treatments, 1) negative control (Control) or 2) water acidifier containing free and buffered SCFA (**SCFA-WA** containing formic acid, acetic acid, propionic acid, copper, zinc acetate, ammonium formate, and a pH of 2.7 to 3.3; Selko B.V., Tilburg, the Netherlands), with 12 pens per treatment. Each room was equipped with two water lines, each connected to a water tank containing the respective treatment. The control group received regular drinking water without any additions. The SCFA-WA group received drinking water with SCFA-WA treatment added to the water tank, where the inclusion rate for the first 3 d was 1 L/1,000L , from days 4 to 6 the inclusion rate was 1.5 L/1,000 L, and from day 7 to the end of the experiment (day 42), the inclusion rate was 2 L/1,000 L. The dosing was based on titration of the drinking water with SCFA-WA until a pH of 3.8 was reached, as per manufacturer recommendations. Once weekly, samples of water were obtained from the drinkers of two pens per treatment per room to monitor pH. One common wheat-based diet was offered to both treatment groups over the two phases, phase 1 being from day 0 to 21 and phase 2 being from days 21 to 42 (Table 1). The feed was formulated to meet nutritional requirements based on commercial guidelines and produced by Target Feeds Ltd. (Whitchurch, UK).

**Table 1.** Feed formulation used in the two feeding phases

Ingredients,%	Phase 1, days 0 to 21	Phase 2, days 21 to 42
Barley raw ground	5.425	3.000
Maize raw ground	8.500	4.500
Wheat raw ground	36.240	56.190
Alphasoy 530	6.375	4.500
Whey powder	6.750	—
Soya hull meal	7.000	6.950
Soya Ext Hipro	10.500	12.650
Full-fat soya Cherwell	12.750	6.750
l-Lysine HCL	0.213	0.225
dl-Methionine	0.128	0.090
l-Threonine	0.085	0.090
Soya oil	2.550	1.485
Limestone Flour Trucal 270	0.850	1.080
Monocalcium phosphate	1.700	1.485
Salt	0.425	0.495
Weaner premix	0.500	0.500
Quantum Blue 5G (ABVista)	0.010	0.010

### Growth performance

Body weight (**BW**) and feed intake were measured on pen level at days 0, 7, 14, 21, 28, 35, and 42. Based on this data, the average daily gain (**ADG**), average daily feed intake (**ADFI**), and feed efficiency (**FE**) were calculated. The water intake was calculated per water line by measuring differences in the level of water in the water tank. To measure the difference in water level, dipsticks were used with 10-L increments. Each morning, the water left in the tank was measured with these dipsticks and rounded up or down to the nearest 10 L. The tanks were subsequently emptied and refilled with fresh water mixed with or without the SCFA-WA. In the afternoon, the water level was checked to make sure the piglets had enough water overnight. Water intake was averaged between the three rooms. Mortality was monitored daily.

### Fecal consistency and sampling

Once daily from days 1 to 14, a visual assessment of the fecal material in each pen was made by three staff members overall, where there was a crossover from one staff member to another, and given a score of 1 to 3 (score 1 = normally shaped feces, score 2 = shapeless (loose) feces, score 3 = thick or thin, liquid feces). The staff members did the scoring sessions together to standardize their interpretations. On days 14 and 42, three average sized piglets per pen, which were representative for the pen, were selected and each used to collect approximately 10 g of feces via rectal palpation. The same piglets were used at both sampling days. Of the three individual samples per pen, two subsamples of at least 1 g each were taken in a cryovial and stored at −80 °C for microbiota analysis. The residual part of the sample was stored at the animal facility as retention sample.

### DNA extraction, polymerase chain reaction, and library preparation

DNA extraction was performed with PowerMicrobiome RNA isolation kit (MO BIO, Carlsbad, CA) following the manufacturer’s instructions with some modifications to extract DNA instead of RNA, such as omitting the β-mercaptoethanol and DNase I steps. The fecal samples were weighted and approximately 70 mg of sample was mixed with 650-µL PM1 solution including 100-µg PureLink RNaseA (Invitrogen, Thermo Fisher Scientific Inc. Hampton, VA). Bacteria in the samples were lysed with MagNA Lyser (Roche, Burges Hill, UK) for 2× 40 s at 5,500 rpm prior to DNA extraction. The DNA was extracted from this cell lysate on the spin filter columns following the manufacturer’s instruction manual, the genomic DNA was eluted from the spin columns in 100 µL 10 mM Tris–HCl buffer (pH 8.0). The concentration of the extracted prokaryotic DNA in each sample was calculated by quantitative polymerase chain reaction (**qPCR**) with 926F (De Gregoris et al., 2011) and 1027R (Claesson et al., 2009) primers at a concentration of 0.4 µM in iQ SYBRgreen Supermix qPCR (Bio-Rad Laboratories Inc., Hercules, CA). PCR for sequencing was carried out in quadruplet replicates with Universal primers 341F-785R of V3–V4 regions to amplify 16S rRNA in a dual-index sequencing strategy according to Kozich et al. (2013) with Taq KAPA HiFi Hotstart ReadyMix (Kapa Biosystems, Woburn, MA) and 12.5 ng bacterial DNA to reduce PCR bias. The cycling conditions were as follows: initial denaturation for 1 min at 95 °C, followed by 25 cycles of denaturation at 95 °C for 20 s, annealing at 56 °C for 30 s, and elongation at 72 °C for 5 min, followed by final elongation for 10 min at 72 °C. Equimolar amounts of the PCR products were pooled for sequencing. The pool was run on an agarose gel, and the amplicon was extracted from the gel and purified by QIAquick Gel Extraction Kit (Qiagen, Hilden, Germany). Negative controls and MOCK communities were included in PCR and the sequencing as controls. The ready-to-load library was sequenced at Eurofins Genomics Europe Sequencing GmbH (Konstanz, Germany) on an Illumina MiSeq Personal Sequencer 2×300 paired end, using the Illumina MiSeq reagent kit.

### Sequencing and bioinformatic data processing

Sequencing reads for each time point were analyzed with NG-Tax pipeline (Ramiro-Garcia et al., 2016) trimming the reads at 150 bp and allowing one mismatch between the reads during OTU picking. Paired end reads were used. Taxonomy was assigned using Silva 138 reference database (Quast et al., 2013). The resulting biom, tree, and metadata files for each time point were uploaded to MicrobiomeAnalyst software (Dhariwal et al., 2017) for further analyses. Samples were rarefied to remove heterogenocity, at equal depth determined by the lowest number of reads of the sample in the set (12,138 reads at day 14 and 6,303 reads on day 42), and relative abundance (**RA**) at each taxonomic level was determined.

### Statistical analysis

Data analysis of the growth performance and fecal scoring was carried out by the statistician of DAH (Stratford-upon-Avon, UK) using GenStat 12.2 (VSN International Ltd, verified Sep 18) software, accepting a level of probability of less than or equal to 0.05 as indicating significance and a level of probability between 0.05 and 0.10 as indicating tendency. Body weight, ADG, ADFI, and FE were analyzed by *t*-test to compare treatments. Body weight, ADG, ADFI, and FE data were analyzed by week, by feeding phase and overall study period. Fecal consistency scores were analyzed by Kruskal–Wallis test per day. Where a significant difference was observed in body weight, ADG, ADFI, FE, or fecal consistency scores, Duncan’s multiple range test was carried out. No outliers were removed for analysis.

Alpha and beta diversity microbiota analyses were performed on rarefied data using MicrobiomeAnalyst. Alpha diversity measures included Chao1 index, Observed Species, Shannon index, and Simpson index and were performed at each taxonomic level data (*T*-test: *P* > 0.05). For the beta diversity analyses, the Bray–Curtis index and Unifrac were used with ANOSIM to assess statistical differences between groups using OUT-level data. The Bray–Curtis index is based on microbial OTU counts and indicates a difference in OTU abundance between treatments (Glen, 2018). The Unifrac measures the difference between collections of 16S sequences as amount of evolutionary history that is unique to them, measured as fraction of branch length in a phylogenetic tree that leads to descendants of one sample. The weighted Unifrac accounts for differences in RAs that can produce different but complementary results (Lozupone et al., 2011). Differentially abundant taxa between treatments at each time point were identified using nonparametric Kruskal–Wallis and Wilcoxon tests in MicrobiomeAnalyst software with false discovery rate (FDR)-corrected *P*-values (*P* < 0.05) to determine significance.

**PCA** (unsupervised principal component analysis), multivariate analysis (redundancy analysis [**RDA**]) with forward selection of the effect of experimental variables (treatment, pen, room, weight on day 0, weigh on day 14, weight on day 42, ADG days 0 to 14, ADG days 14 to 42, ADG days 0 to 42, ADFI days 0 to 14, ADFI days 14 to 42, ADFI days 0 to 42, FE days 0 to 14, FE days 14 to 42, FE days 0 to 42, average diarrhea score days 0 to 14), and the partial RDA to evaluate the effect of experimental variables separately were performed in Canoco5 (Lepš and Šmilauer, 2014). In the RDA plots, 20 best-fitting genera (or highest possible taxonomic rank assigned) were displayed, and the arrows corresponding to these taxa point toward environmental variables that were positively correlated with these microbial groups.

## Results

### Growth performance

Growth performance was measured and analyzed weekly and per feeding phase. Due to limited differences on weekly basis, only the data per feeding phase were shown. Growth performance results were summarized in Table 2. No significant improvement was shown in body weight, ADG, or ADFI when piglets were provided SCFA-WA. There was a strong tendency for improved FE in the first feeding phase (days 0 to 21) in the SCFA-WA group compared with control (*P* = 0.055). FE was significantly improved in SCFA-WA (0.696) compared with the control (0.650; *P* = 0.025, data not shown) in the third week of the study, which likely contributed to the strong tendency observed in the first feeding phase. Over the entire study (days 0 to 42), FE was significantly improved in SCFA-WA piglets (*P* = 0.042). During the second feeding phase (days 21 to 42) and over the whole study period (days 0 to 42), the SCFA-WA group consumed significantly more water (*P* = 0.028 and *P* = 0.043, respectively; Table 2). The pH of drinking water was measured throughout the study, where pH of the control group ranged from 6.89 to 8.03, whereas the pH in the SCFA-WA group was between 2.90 and 3.79. There was no mortality over the course of the study.

**Table 2.** Body weight (BW), average daily gain (ADG), average daily feed intake (ADFI), feed efficiency (FE), and average water intake (AWI) of piglets provided with control water or water with water acidifier containing free and buffered SCFA (SCFA-WA)

	Day	Control	SCFA-WA	SED^1^	P-value
BW, kg	0	8.74	8.72	0.437	0.955
	21	14.49	14.65	0.501	0.741
	42	28.11	28.93	0.797	0.317
ADG, kg/piglet/day	0–21	0.274	0.283	0.0162	0.576
	21–42	0.649	0.680	0.0192	0.121
	0–42	0.461	0.481	0.0142	0.172
ADFI, kg/piglet/day	0–21	0.392	0.382	0.0181	0.585
	21–42	1.070	1.114	0.0299	0.162
	0–42	0.731	0.748	0.0196	0.406
FE	0–21	0.697	0.738	0.0203	0.055
	21–42	0.607	0.610	0.0054	0.514
	0–42	0.631^a^	0.643^b^	0.0057	0.042
AWI, L/pen	0–21	17.08	18.18	0.985	0.327
	21–42	25.53^a^	30.00^b^	1.324	0.028
	0–42	21.26^a^	24.09^b^	0.966	0.043

^1^SED, the standard error of the difference.

^a,b^Values with a different superscript within a row differ significantly (*P* < 0.05).

### Fecal consistency score

The sum of total incidence of feces with score 1 (no diarrhea) was numerically higher in the control group (Control = 32, SCFA-WA = 28), whereas feces with the score 3 (diarrhea) was numerically higher in the treatment with SCFA-WA (Control = 75, SCFA-WA = 81). Whether piglets in the treatment with SCFA-WA had more diarrhea cannot be concluded as statistical analysis was not performed over the total sum per treatment. On day 13, there was a significant difference in fecal scoring (Mean ranking: Control = 15, SCFA-WA = 10, *P* = 0.045); however, the overall differences were not significant (data not shown). In general, the diarrhea incidence was relatively low throughout this study.

### Microbiota

A total of 1,919,530 sequencing reads were obtained from the 72 samples from day 14 and 21,216,893 reads from the 72 samples collected on day 42. Rarefaction cutoff values were based on the minimal number of reads per sample and were set to 12,138 counts for day 14 and 6,303 for day 42.

On day 14, ANOSIM analysis showed a significant difference between control and SCFA-WA treatment in beta diversity Bray–Curtis index (*P* = 0.024), but there was no difference in the unweighted and weighted Unifrac. On day 42, there was a significant difference in the Bray–Curtis index and weighted Unifrac between the two treatments (*P* = 0.002 and *P* = 0.003). On day 14, *T*-test statistics showed that none of the alpha diversity metrices differed between the treatment groups. On day 42, Shannon and Simpson indexes at Class level were significantly lower in the SCFA-WA treatment (*P* = 0.047 and *P* = 0.018) and Shannon and Simpson indexes at Order level were significantly higher in the SCFA-WA treatment (*P* = 0.029 and *P* = 0.024).

On day 14, univariate statistics using Kruskal–Wallis test showed no significant differences in taxonomy between the treatments when using the FDR-adjusted *P*-values. However, the unadjusted *P*-value showed differences in taxonomy between the treatments at Genus, Family, Order, Class, and Phylum level in taxa with varying prevalence in animals in each treatment as indicated in Table 3. At Genus level, SCFA-WA had a higher RA of *Clostridium sensu stricto 1*, *Streptococcus*, *Libanicoccus*, *Fournierella*, *Lachnoclostridium*, and lower RA of *Prevotellaceae UCG 003*, *Dialister* (*P* < 0.05), and a trend in *Bacteroides pectinophilus* group (*P* = 0.054) and *Eubacterium nodatum* group (*P* = 0.059). At Family level, a higher RA was seen in Streptococcaceae and Clostridiaceae and a lower RA of unassigned family (Table 3). On day 42, the adjusted *P*-value (FDR) showed a significantly higher RA of *Clostridium sensu stricto 1* and lower RA of *Streptococcus* at Genus level for the treatment for the treatment with SCFA-WA (Table 4). At Family level, there was a significantly higher RA in SCFA-WA of Clostridiaceae and lower RA of Streptococcaceae; at Order level, higher levels of Clostridiales and Gastranaerophilales and a lower level of Lactobacillales; and at Class lower levels of *Clostridia* and *Bacilli*. In addition, the uncorrected *P*-value indicated the presence of additional differentially abundant taxa between treatments (Table 4).

**Table 3.** Relative abundance (RA) and prevalence of the genera, family, order, and class with a significant (*P* < 0.05, FDR adjusted and unadjusted) difference in response to the water treatment with water acidifier containing free and buffered SCFA (SCFA-WA) at day 14

Day 14	Control		SCFA-WA		Total samples		Kruskal–Wallis	
	Average RA	Prevalence (*n* = 36)	Average RA	Prevalence (*n* = 36)	Average RA	Prevalence (*N* = 72)	FDRp	*P*-value
*g__Prevotellaceae_UCG-003*	↑0.0022	23	↓0.0014	14	0.0018	37	0.566	0.036
*g__Clostridium_sensu_stricto_1*	↓0.0006	5	↑0.0027	13	0.0016	18	0.566	0.031
*g__Streptococcus*	↓0.0002	3	↑0.0037	11	0.0020	14	0.566	0.013
*g__[Bacteroides]_pectinophilus_group*	↑0.0014	9	↓0.0005	4	0.0010	13	0.566	0.054
*g__[Eubacterium]_nodatum_group*	↑0.0014	8	↓0.0011	4	0.0013	12	0.566	0.060
*g__Libanicoccus*	↓0.0001	2	↑0.0003	7	0.0002	9	0.566	0.023
*g__Fournierella*	↓0.0001	1	↑0.0005	7	0.0003	8	0.566	0.029
*g__Lachnoclostridium*	↓0.0000	0	↑0.0005	7	0.0003	7	0.566	0.006
*g__Dialister*	↑0.0015	4	↓0.0000	0	0.0007	4	0.566	0.043
*f__Clostridiaceae*	↓0.0006	6	↑0.0031	14	0.0019	20	0.690	0.031
*k__NA;f__NA*	↑0.0004	6	↓0.0001	1	0.0003	7	0.706	0.047
*f__Streptococcaceae*	↓0.0002	3	↑0.0037	11	0.0020	14	0.568	0.013
o__Clostridiales	↓0.0006	6	↑0.0031	14	0.0019	20	0.828	0.031
c__NA	↑0.0004	6	↓0.0001	1	0.0003	7	0.352	0.022
*p__NA*	↑0.0004	6	↓0.0001	1	0.0003	7	0.584	0.049

**Table 4.** Relative abundance (RA) and prevalence of the genera, family, order, and class with a significant (*P* < 0.05, FDR adjusted and unadjusted) difference in response to the water treatment with water acidifier containing free and buffered SCFA (SCFA-WA) at day 42

Day 42	Control		SCFA-WA		Total		Kruskal–Wallis	
	Average RA	Prevalence (*n* = 36)	Average RA	Prevalence (*n* = 36)	Average RA	Prevalence (*N* = 72)	FDRp	*P*-value
*g__Clostridium_sensu_stricto_1*	↓0.0850	36	↑0.1471	36	0.1161	72	0.020	0.000
*g__Streptococcus*	↑0.1028	36	↓0.0444	34	0.0736	70	0.020	0.000
*g__Turicibacter*	↓0	0	↑0.0018	8	0.0009	8	0.107	0.003
*g__Eubacterium eligens group*	↓0.0016	21	↑0.0029	28	0.0022	49	0.207	0.008
*g__Lachnospira*	↓0.0000	1	↑0.0004	9	0.0002	10	0.325	0.015
*g__Lachnospiraceae_UCG_008*	↓0	0	↑0.0006	5	0.0003	5	0.388	0.022
*g__Candidatus Saccharimonas*	↓0.0003	6	↑0.0010	13	0.0006	19	0.448	0.033
*g__Fournierella*	↓0.0008	12	↑0.0015	22	0.0012	34	0.448	0.036
*g__Ligilactobacillus*	↑0.0005	4	↓0	0	0.0002	4	0.448	0.043
*g__Lachnospiraceae_NC2004_group*	↑0.0004	6	↓0.0001	3	0.0003	9	0.448	0.043
*f__Streptococcaceae*	↑0.1028	36	↓0.0444	34	0.0736	70	0.008	0.000
*f__Clostridiaceae*	↓0.0852	36	↑0.1472	36	0.1162	72	0.008	0.000
*o__Gastranaerophilales; f__uncultured_bacterium*	↓0.0001	2	↑0.0007	10	0.0004	12	0.156	0.011
*f__Erysipelotrichaceae*	↓0.0049	29	↑0.0078	35	0.0063	64	0.287	0.030
*f__Saccharimonadaceae*	↓0.0003	6	↑0.0010	13	0.0006	19	0.287	0.033
*o__Coriobacteriales;f__uncultured*	↓0.0001	3	↑0.0004	7	0.0002	10	0.321	0.045
*o__Clostridiales*	↓0.0852	36	↑0.1472	36	0.1162	72	0.009	0.000
*o__Lactobacillales*	↑0.3095	36	↓0.2056	36	0.2575	72	0.018	0.001
*o__Gastranaerophilales*	↓0.0001	2	↑0.0007	10	0.0004	12	0.048	0.006
*o__Erysipelotrichales*	↓0.0073	30	↑0.0107	35	0.0090	65	0.129	0.022
*o__Clostridia_UCG_014*	↓0.0089	35	↑0.0135	35	0.0112	70	0.131	0.027
*o__Saccharimonadales*	↓0.0003	6	↑0.0010	13	0.0006	19	0.133	0.033
*c__Clostridia*	↑0.0834	36	↓0.0730	36	0.0782	72	0.005	0.000
*c__Bacilli*	↑0.1033	36	↓0.0950	36	0.0992	72	0.009	0.001
*c__Vampirivibrionia*	↓0.0002	2	↑0.0009	10	0.0006	12	0.026	0.006
*c__Saccharimonadia*	↓0.0004	6	↑0.0013	13	0.0009	19	0.108	0.033
p__Cyanobacteria	↓0.0001	2	↑0.0007	10	0.0004	12	0.066	0.006
p__Patescibacteria	↓0.0003	6	↑0.0010	13	0.0006	19	0.183	0.033

**Figure 1.**
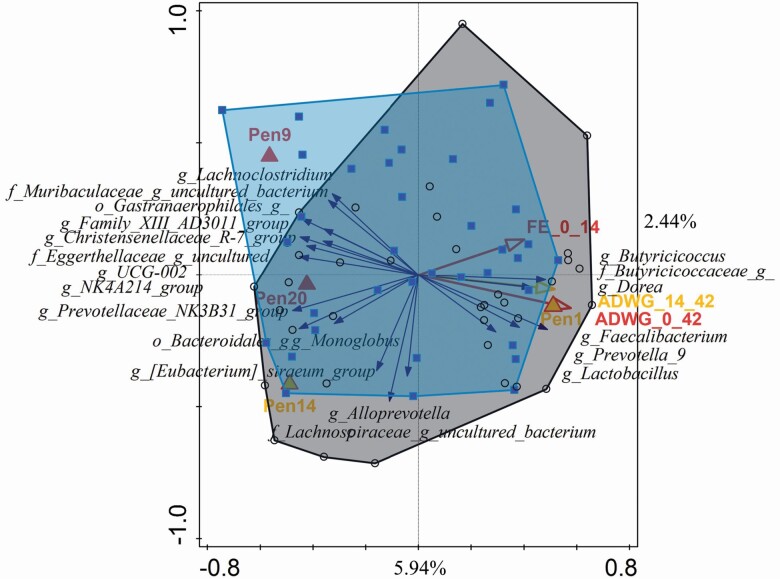
Redundancy analysis (RDA) on genus level at day 14. Pigs of the control group are included in the gray area, and the pigs with the water acidifier containing free and buffered SCFA (SCFA-WA) are included in the blue area. The red arrows represent a significant (*P* < 0.05) relationship, and yellow arrows represent a tendency (0.05 < *P* < 0.10).

PCA indicated no separation in microbiota communities between the treatments or room. When using forward selection, the selected variables explained 14.64% of total variation with most variation explained by average daily weight gain between days 0 to 42 (2.7%; *P* = 0.018), FE 0 to 14 (2.0%; *P* = 0.048), and several pens. The taxa most related to the average daily weight gain and FE were *Lactobacillus* and *Butyricicoccus*, unknown genus in Butyricicoccaceae family, *Prevotella 9*, and *Faecalibacterium* (Figure 1).

No link between the microbiota at day 14 and the incidence of diarrhea could be made, as microbiota was measured at an individual level, whereas diarrhea was measured at pen level.

**Figure 2.**
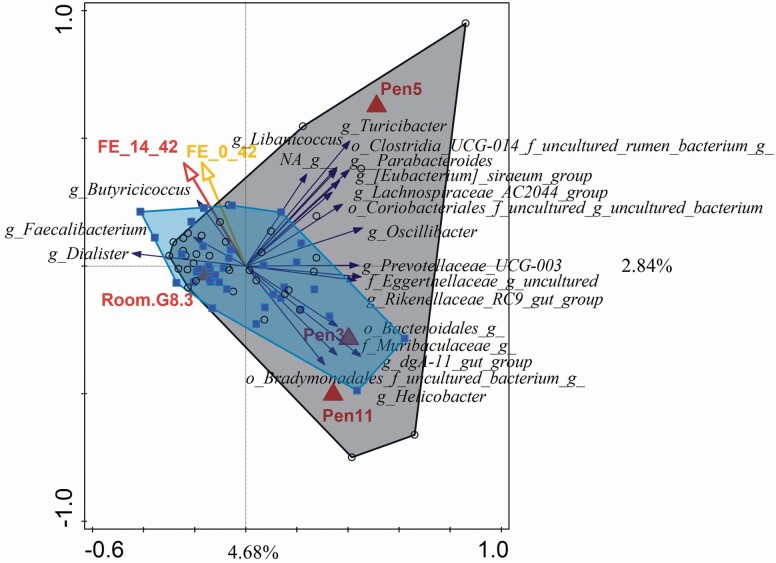
Redundancy analysis (RDA) on genus level at day 42. Pigs of the control group are included in the gray area, and the pigs with the water acidifier containing free and buffered SCFA (SCFA-WA) are included in the blue area. The red arrows and triangles represent a significant (*P* < 0.05) relationship, and yellow arrows represent a tendency (0.05 < *P* < 0.10).

PCA of day 42 indicated no clear separation of samples with respect to treatment, room, or pen. When forward selection was used to select best subset of variables summarizing variation at genus-level microbiota composition, a room, several pens, and FE between days 14 and 42 were selected and explained 14.13% of total variation (Figure 2).

RDA showed that control and SCFA-WA explained 1.82% (*P* = 0.068 and *P* = 0.064, respectively). On day 42, *Butyricicoccus* and *Faecalibacterium* seemed to be associated with greater FE, similar to the observation made on day 14.

## Discussion

The SCFA-WA used in the current study reduced the pH of drinking water from 6.89–8.03 to 2.90–3.79 and improved FE in piglets at weaning. During weaning, the stomach pH in piglets increases, resulting in less activity of the enzyme pepsin as well as less stimulation of the secretion of pancreatic enzymes (Partanen and Mroz, 1999; Nowak et al., 2021). This could lead to less-efficient digestion of nutrients (Partanen and Mroz, 1999; Heo et al., 2013; Nowak et al., 2021) and therefore a lower FE. When included in water acidifiers, organic acids may lower the pH in the stomach (Suiryanrayna and Ramana, 2015), suggesting potential use for improvements in growth performance. Therefore, an improved FE is expected when SCFA-WA are administered. There is a paucity of data available on the effect of water acidifiers on growth performance. De Busser et al. (2011) showed that with a blend of lactic, formic, propionic, and acetic acid in different doses added to the drinking water of weaned piglets, there were no differences in feed intake, weight gain, final body weight, or FE, although numerically the FE was improved with the highest dose of water acidifier. Walsh et al. (2007) on the other hand showed that when piglets received a blend of mostly propionic, acetic, and benzoic acid, there was an improvement in weight gain and feed intake in the last phase of postweaning (days 21 to 34); however, over the whole postweaning period (days 0 to 34), a decrease in FE (0.017) was shown. In the present study, the piglets with SCFA-WA had a significantly improved FE, which is line with De Busser et al. (2011). Furthermore, although not significant, the piglets with SCFA-WA had a marginally higher body weight and ADG, which is in line with Walsh et al. (2007). Nutrient digestibility and stomach pH levels were not measured; therefore, further research is needed to provide evidence of this suggested mode of action of the SCFA-WA. The SCFA-WA group also had a significant increase in water consumption, suggesting improved palatability, in line with Nowak et al. (2021), who report enhanced smell and palatability with the use of water acidifiers. Increased water intake is often associated with higher feed intake (De Busser et al., 2011), although this was not observed in the current study. Furthermore, Walsh et al. (2007) also reported an increase in water intake of 47% when supplemented with water acidifiers, whereas De Busser et al. (2011) showed a negative effect up to 26% on water intake when water acidifiers were provided. The reason why in some studies water intake is increased and in other studies is decreased is unknown. This might be influenced by the specific acids used in the blends as well as the doses.

There was a significantly higher RA of *Clostridium sensu stricto 1* and lower RA of *Streptococcus* at Genus level when piglets were supplemented with SCFA-WA. *Clostridium sensu stricto 1* belong to the *Clostridiaceae*. He et al. (2019) showed that *Clostridiaceae* was more abundant in pigs with a higher FE in a feed intake model in which piglets with the same ADG were divided in two groups based on feed intake. In the current study, the piglets that received SCFA-WA had a higher FE along with a higher RA of members of the *Clostridiaceae* taxa. Furthermore, some members of *Clostridium* can produce SCFAs by consuming mucus-derived saccharides as energy source. This in turn has a beneficial effect on intestinal mucosa barrier, which leads to the possible inhibition of pathogen adherence (Wlodarska et al., 2015; He et al., 2020). In the current study, the SCFA production in the gut was not measured and to further substantiate this mode of action, additional research is needed.

*Streptococcus* belong to the lactic acid bacteria. Some species have probiotic characteristics (Zhou et al., 2016; Fernandez et al., 2018); however, they are generally associated with pathogenic bacteria (Köhler, 2007; Moreno et al., 2016). The most important pathogenic species for the pig industry is *Streptococcus suis*, which is the cause of various diseases such as meningitis, septicemia, and endocarditis (Moreno et al., 2016; Murase et al., 2019). Although *S. suis* mainly infect piglets through the upper respiratory tract, studies have shown that the gastrointestinal tract of weaned piglets is rapidly colonized by *S. suis* (Su et al., 2008; Ferrando et al., 2015). The gastrointestinal tract can become the entry point via which it can infect piglets, especially during periods of high stress (Swildens et al., 2004; Ferrando et al., 2015). As multiple *Streptococcus* species could cause disease, a lower abundance of *Streptococcus* could be considered beneficial for the animal. In the current study, the SCFA-WA showed a lower abundance in *Streptococcus*. As no species identification was possible with the current method, we cannot conclude whether the lower abundance of *Streptococcus* observed here were commensal or pathogenic species.

The RDA of the fecal samples showed a positive correlation between improved FE and daily weight gain and higher RA of genera *Butyricicoccus* and *Faecalibacterium*. Although no species could be assigned in this study, the aforementioned genera are known to include species that could impart performance and health benefits, particularly via the production of butyrate.

One of the most known *Faecalibacterium* is *F. prausnitzii*, known as a beneficial bacterium which shows anti-inflammatory effects due to the secretion of metabolites blocking NF-κB activation and IL-8 secretion as well as decreasing proinflammatory cytokine synthesis and increasing anti-inflammatory cytokine secretion (Sokol et al., 2008; Miquel et al., 2013). *Faecalibacterium*, like *Butyricicoccus*, belong to the butyrate producers (Kubasova et al., 2018). A higher abundance of *Faecalibacterium* is expected to be beneficial for the animal, as in humans, a low abundance of *Faecalibacterium* is often associated with inflammatory bowel disease (Miquel et al., 2013).

The butyrate-producing *Butyricicoccus pullicaecorum* has previously been used as probiotic in broilers (Eeckhaut et al., 2016). The authors report beneficial effects of the probiotic on feed conversion ratio, a phenotypic response that concurs with the finding of the current study. In grower/finisher pigs, *Butyricicoccus pullicaecorum* seems to be enhanced in response to a corn-soybean diet, probably due to its starch-degrading capacities (Verschuren et al., 2018). This latter study suggests that the influence of microbiota on the FE may be due to an effect of SCFA produced in the gut. Although in the current study the species and strain of *Butyricicoccus* is not known, it is possible that the genus members included butyric acid producing organisms that had an effect via volatile fatty acid production.

Most of the bacterial genera found to be associated with higher FE are described as butyrate producers. Butyric acid functions as the main source of energy for colonocytes (Suiryanrayna and Ramana, 2015; Nowak et al., 2021). It further promotes proliferation and differentiation of intestinal cells (Suiryanrayna and Ramana, 2015; Gresse et al., 2017; Nowak et al., 2021). Kubasova et al. (2018) showed a higher abundance of *Faecalibacterium*, associated with higher feed intake. However, in this study, fecal SCFA including butyrate, were not measured; thus, this hypothesis needs to be further investigated.

In conclusion, piglets provided with SCFA-WA had a significantly improved FE and higher water intake compared with controls. There was no significant difference in body weight, ADG, or ADFI. Furthermore, there was no overall significant difference in fecal scoring between the treatments. Piglets provided with SCFA-WA showed a significantly higher RA of *Clostridium sensu stricto 1* and lower RA of *Streptococcus*. Furthermore, the RDA showed a positive association between improved FE and RA of *Butyricicoccus* and *Faecalibacterium* known to include butyrate-producing species considered to be beneficial for the health of the animals. The results from this study demonstrate that providing water acidifier containing free and buffered SCFA to newly weaned piglets can modulate the microbiota and improve FE in piglets.

## Abbreviations

ADFI: average daily feed intake
ADG: average daily gain
AWI: average water intake
BW: body weight
FDR: false discovery rate
FE: feed efficiency
PCA: principal component analysis
PCR: polymerase chain reaction
qPCR: quantitative polymerase chain reaction
RA: relative abundance
SCFA: short-chain fatty acids
WA: water acidifier
